# Recovering from a renal vascular catastrophe: Case report 

**DOI:** 10.5414/CNCS110984

**Published:** 2023-03-05

**Authors:** Diogo Francisco, Gonçalo Pimenta, Ana Cristina Martins, Ivo Laranjinha, Hermínia Estibeiro, Célia Gil, Margarida Gonçalves, Maria Augusta Gaspar

**Affiliations:** Hospital de Santa Cruz, Centro Hospitalar Lisboa Ocidental, Lisboa, Portugal

**Keywords:** renal artery thrombosis, renal infarction, acute kidney injury

## Abstract

Renal artery thrombosis is a rare vascular event that precipitates renal infarction. Although in up to one third of cases the etiology is not identified, renal artery lesions, cardioembolism and acquired thrombophilias are the main causes. A bilateral simultaneous idiopathic renal artery thrombosis is an unlikely coincidence. We present two cases of patients with acute bilateral renal artery thrombosis of unknown etiology. Cardiac embolism, acquired thrombophilia and occult neoplasm workups were negative. Both cases were temporarily hemodialysis-dependent and partially recovered renal function under conservative approach with systemic anticoagulation. Recommendations on optimal treatment for renal artery thrombosis are still lacking. We discuss the available options.

## Background 

Acute renal artery thrombosis (ARAT) is a rare and catastrophic cause of renal infarction (RI). Etiologies for RI have been classified in three major categories – renal artery lesion, cardioembolism, and hypercoagulable states. In situ thrombosis has been observed as a consequence of atherosclerotic disease or structural vascular lesions such as fibromuscular dysplasia; infrequently, thrombosis can be due to thrombophilia, such as antiphospholipid syndrome, inflammatory conditions, such as large vessel vasculitis, infectious diseases such as syphilis, or sickle cell disease [1]. As discussed below, optimal treatment strategies recommendations are still lacking. We present two cases of bilateral ARAT that recovered kidney function on a conservative approach with systemic anticoagulation and review the clinical findings and therapy options described in the literature until this time. 

## Case 1 

**Figure 1:**
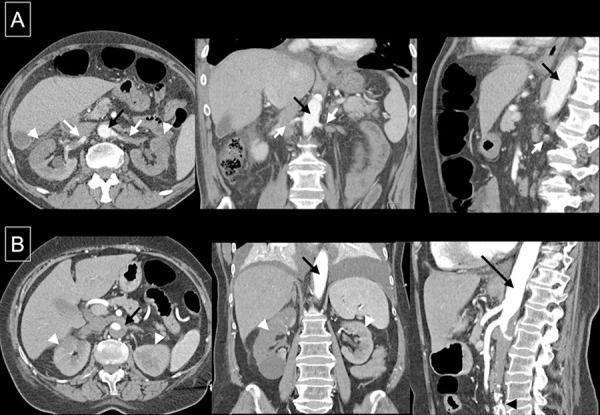
A: CTA images at diagnosis of Case 1. B: CTA images at diagnosis of Case 2. White arrows = renal artery; black arrows = aorta; white arrowheads = renal infarct; black arrowheads = aortoiliac stent.

A 73-year-old male with no history of cardiac disease, hypertension, or recent trauma was admitted with right flank pain and vomiting. He had a baseline serum creatinine (sCr) of 1.2 mg/dL 6 months before. At hospital admission, he was severely hypertensive (204/103 mmHg) and had anuric acute kidney injury (AKI) with elevated lactate dehydrogenase (LDH). He immediately started hemodialysis. Renal computerized tomography angiogram (CTA) performed at 48 hours of symptom onset showed bilateral arterial thrombosis – left renal artery totally occluded and partial occlusion of the right renal artery with slight enhancement of the corresponding kidney (Figure 1A). There were no signs of arterial dissection. 

Anticoagulation with low molecular weight heparin (LMWH) was started (enoxaparin 1 mg/kg/day). An echocardiogram and Holter monitoring showed no abnormalities; thorax and abdomen computerized tomography (CT), upper endoscopy, and colonoscopy showed no occult neoplasm; and a full panel for acquired thrombophilias was negative (detailed in Discussion). 

He was maintained on warfarin therapy (INR goal of 2 – 3) and progressively recovered kidney function, becoming independent from dialysis 54 days after onset. CTA repeated 7 days after initiation of anticoagulation showed partial re-permeabilization of the right artery but no improvement in the left kidney perfusion. After 18 months, he was at stage 3b chronic kidney disease (CKD) with serum creatinine of 1.87 mg/dL. 

## Case 2 

A 65-year-old female was admitted with dyspnea associated with right lumbar pain. She was severely hypertensive (228/138 mmHg), had peripheral edema and mild hypoxemia (PaO_2_ 62 mmHg and O_2_ saturation 92%). Her prior history included heavy smoking and atherosclerotic artery disease. She had an aortoiliac stent placed 20 years before; she was on clopidogrel. She had no history of atrial fibrillation or cardiac insufficiency. 

Her laboratory results at admission showed normal kidney function (sCr 0.98 mg/dL, urea 60 mg/dL) with elevated LDH (309 U/L) and normal proBNP (3,625 pg/mL). Urinalysis showed hematoproteinuria. 

A contrast-enhanced CT scan showed bilateral pleural effusion and excluded pulmonary embolism. She was admitted with fluid overload, attributed to previously undiagnosed cardiac insufficiency, and was started on diuretics. Her echocardiography showed mildly depressed ejection fraction and inferior vena cava with inspiratory collapse. Maintaining lumbar pain, abdominal ultrasound and CT scan were unremarkable. She was started on intravenous nitrates for hypertension control. After 24 hours of admission, anuria was verified. Due to refractory fluid overload, she was admitted in the intensive care unit and started on hemodialysis. 

In a renal CTA performed 96 hours after symptom onset, a large aortic thrombus that occluded both renal arteries was detected (Figure 1B). The main right renal artery was occluded, but the kidney had partial reperfusion through a polar accessory artery. The left renal artery was totally occluded. 

The patient was started on conservative therapy with enoxaparin (1 mg/kg/day). After clinical stabilization, anticoagulation was switched to warfarin (INR goal 2 – 3). Echocardiogram and Holter monitoring showed no sign of embolic source; thorax and abdomen CT, upper endoscopy, and colonoscopy showed no underlying neoplasm; a complete panel for acquired thrombophilias was negative (detailed in Discussion). CTA was not repeated. Under anticoagulation, she progressively recovered renal function and was independent from hemodialysis 30 days after symptom onset. Twelve months later, she was at stage 4 CKD with sCr 2.81 mg/dL. 

## Discussion 

### Diagnosis 

RI may represent a diagnostic trap, in which early treatment may impact prognosis. Nevertheless, a non-specific constellation of symptoms associated with AKI may prevent physicians from requiring diagnostic CTA with contrast administration. Diagnosis is often delayed, with unpredictable consequences for future kidney function. A high index of suspicion is therefore required. Hallmarks of disease have long been identified in multiple series. Lumbar pain is almost invariably present, associated with AKI, severe renin-mediated hypertension, and elevated cell necrosis marker LDH [1, 2, 3]. Case 2 eloquently represents a diagnostic odyssey of a renal vascular catastrophe. If a RI develops bilaterally or in a single kidney, anuria also becomes a clinical feature. In most of the cited literature, unilateral RI cases are dominant. The cause for RI remained unknown in up to a third of patients [1, 4, 5, 6]. Since they are two noncontinuous vascular territories, bilateral idiopathic renal artery thrombosis is an unlikely coincidence. 

COVID-19 has been associated with thrombotic events [7], as well as rare cases of thrombotic events following vaccination [8]. Neither of the two cases presented had recent SARS-CoV-2 vaccination or infection. 

Cardiac disease – especially atrial fibrillation – is an important source for embolism [1, 5, 6, 9, 10, 11] that ought to be excluded. Systemic hypercoagulable states usually affect multiple vascular territories; therefore, acquired thrombophilias must also be searched for [12, 13]. These are antiphospholipid syndrome, protein C and S deficiency, factor V Leiden, antithrombin III deficiency, hyperhomocysteinemia, prothrombin gene mutation. For etiologic search purposes both case 1 and 2 underwent workup with Holter monitoring, echocardiography, full thrombophilia panel, and occult neoplasm search. Although she had significant atherosclerotic risk factors, the cause of the primary aortoiliac artery disease in case 2 is not totally clear; bilateral renal artery thrombosis seems to be the consequence of stent thrombosis with proximal extent. Case 1 is an example of an idiopathic bilateral renal artery thrombosis that was negative for an extensive study of secondary causes. 

### Treatment 

As a rare condition, the evidence for optimal management for ARAT is scarce. Guidelines and recommendation are lacking, and great disparities in treatment approach have been identified. Anticoagulation is the cornerstone of RI treatment. A recent retrospective descriptive analysis of patients with acute RI demonstrated successful early outcomes with dialysis-free survival at 1 year of 91%, but a drop to 64% at 5 years. All patients were put on conservative single anticoagulation therapy [14], however only a minority (6%) had bilateral RI. Although there is a consensus on the benefit of anticoagulation, there are still many questions remaining: there are no available data on the efficacy and safety profiles of new oral anticoagulants; there is no established role for antiaggregation therapy, and duration of anticoagulation is also undefined (Table 1). As an example, should an idiopathic renal artery thrombosis remain on anticoagulation of indefinite duration, as is recommended for pulmonary embolism with no identified risk factor [15]? 

Surgical embolectomy has been associated with an unacceptable mortality of 5.5% [16], and systemic fibrinolysis has not demonstrated clinical benefit [17]. Endovascular procedures for renal artery thrombosis encompass catheter-directed thrombolysis (CDT), pharmacomechanical thrombectomy, and aspiration thrombectomy with possible angioplasty. Endovascular procedures should be reserved for patients who do not have prolonged ischemia time with no cortical atrophy and no contraindication to fibrinolytics [18]. CDT is an established alternative to surgery in acute limb ischemia [19], and successful cases have been described in ARAT [20, 21, 22, 23, 24]. Bleeding complications must be anticipated and weighed against benefits. Long-term benefits are difficult to evaluate because in most cases the contralateral kidney artery was not involved. Endovascular thrombectomy has also successfully been performed [25, 26]. More recent works performing aspiration thrombectomy with or without subsequent local fibrinolysis provided good outcomes [27, 28]. The choice between surgical or endovascular therapy should consider each center’s experience and expertise. 

The timeframe for endovascular therapy of other vascular diseases such as stroke and acute coronary syndrome is well established [15, 29]. Controversies persist on the clinical benefit of invasive treatment for ARAT with delayed diagnosis. Animal studies have demonstrated great renal parenchymal loss after 1 hour of ischemia [30, 31, 32]. However, these studies fail to report on the kidney regeneration capacity after acute tubular necrosis and the role of collateral vascular supply. Even though RI is established after 8 hours of ischemia, successful revascularizations have been described after this period [16, 26, 33]. Renal collateral vascular supply seems to partially sustain renal viability. Both cases 1 and 2 were proposed for intervention, but according to our vascular team’s point of view, renal artery thrombosis with more than 24 hours from onset induces permanent damage to the kidney. Therefore, any intervention other than antithrombotic therapy was considered to be futile, and the risks outweighed the potential benefits. Looking back, one may consider there was a recovery potential that was probably underappreciated. 

## Conclusion 

As previously discussed, in cases of ARAT, diagnostic delay is frequent and may prevent timely treatment. It is currently not clear which patients could benefit from endovascular procedures and in what timeframe do they still benefit. Even though there was no return to baseline kidney function in both cases 1 and 2, one may argue whether we might expect better outcomes after such insult with more invasive procedures. Recovering from a renal vascular catastrophe will not come without sequelae, which will certainly impact glomerular filtration rate if it affects the whole kidney parenchyma. 

## Acknowledgment 

We thank the medical team of Nephrology Department at Hospital Santa Cruz and Intensive Care Unit at Hospital São Francisco Xavier. 

We thank the medical team of Vascular Surgery Department at Hospital Egas Moniz. 

## Funding 

This research received no funding. 

## Conflict of interest 

None to declare. 

**Table 1.** Remaining questions in the treatment of renal artery thrombosis.

Remaining questions in the treatment of renal artery thrombosis
What is the efficacy of the new oral anticoagulants? Is there a benefit with antiplatelet therapy? What is the optimal duration of therapy? What is the best timing for endovascular procedure
